# Correction: Corsaro et al. Notch, SUMOylation, and ESR-Mediated Signalling Are the Main Molecular Pathways Showing Significantly Different Epimutation Scores between Expressing or Not Oestrogen Receptor Breast Cancer in Three Public EWAS Datasets. *Cancers* **2023**, *15*, 4109

**DOI:** 10.3390/cancers16112037

**Published:** 2024-05-28

**Authors:** Luigi Corsaro, Davide Gentilini, Luciano Calzari, Vincenzo Sandro Gambino

**Affiliations:** 1 Department of Brain and Behavioral Sciences, Università di Pavia, 27100 Pavia, Italy; davide.gentilini@unipv.it (L.C.); vincenzo.gambino@aslto3.piemonte.it (V.S.G.); 2 Centro Diagnostico Italiano, Dipartimento di Genetica Molecolare, 20100 Milano, Italy; 3 Bioinformatics and Statistical Genomics Unit, IRCCS Istituto Auxologico Italiano, 20145 Milan, Italy; l.calzari@auxologico.it; 4 Ospedale di Rivoli, 10098 Rivoli, Italy

**Additional Affiliation(s)**

In the published article [1], there was an error regarding the affiliations for Luigi Corsaro and Vincenzo Sandro Gambino.

The correct affiliations are listed below:

Luigi Corsaro ^1,2,†^

^1^ Department of Brain and Behavioral Sciences, Università di Pavia, 27100 Pavia, Italy

^2^ Centro Diagnostico Italiano, Dipartimento di Genetica Molecolare, 20100 Milano, Italy

Vincenzo Sandro Gambino ^1,4,†^

^1^ Department of Brain and Behavioral Sciences, Università di Pavia, 27100 Pavia, Italy

^4 ^ Ospedale di Rivoli, 10098 Rivoli, Italy

The authors apologize for any inconvenience caused and state that the scientific conclusions are unaffected. This correction was approved by the Academic Editor. The original publication has also been updated.

**Addition of Authors**

Davide Gentilini and Luciano Calzari were not included as authors in the original publication. The corrected Author Contributions Statement is as follows:

**Author Contributions:** Conceptualization, L.C. (Luigi Corsaro) and V.S.G.; Methodology, L.C. (Luigi Corsaro), D.G., L.C. (Luciano Calzari) and V.S.G.; Validation, D.G. and L.C. (Luciano Calzari); Software, L.C. (Luigi Corsaro), D.G. and L.C. (Luciano Calzari); Formal analysis, L.C. (Luigi Corsaro) and V.S.G.; Investigation, V.S.G.; Resources, L.C. (Luigi Corsaro) and V.S.G.; Data curation, L.C. (Luigi Corsaro); Writing—original draft, L.C. (Luigi Corsaro) and V.S.G.; Writing—review and editing, L.C. (Luigi Corsaro) and V.S.G.; Visualization, L.C. (Luigi Corsaro); Supervision, L.C. (Luigi Corsaro), D.G., L.C. (Luciano Calzari) and V.S.G. All authors have read and agreed to the published version of the manuscript.

The authors apologize for any inconvenience caused and state that the scientific conclusions are unaffected. This correction was approved by the Academic Editor. The original publication has also been updated.
